# Supplementary material for: Systematic Review and Meta-Analysis of Sex-Specific COVID-19 Clinical Outcomes
Source: Front Med (Lausanne). 2020 Jun 23;7:348. doi: 10.3389/fmed.2020.00348 (PMC7331754; doi:10.3389/fmed.2020.00348)
Supplement: Supplementary file 1 [file Data_Sheet_1.PDF]

Systematic Review and Meta-Analysis of Sex-Specific COVID-19 Clinical Outcomes

Supplemental Data 1

```
##### R Scripts for COVID-19 Meta-Analysis

##### References
########################################################################
#Overview of R Tools: https://cran.r-project.org/web/views/MetaAnalysis.html
#Package: https://cran.r-project.org/web/packages/meta/meta.pdf
#Book (free): https://bookdown.org/MathiasHarrer/Doing_Meta_Analysis_in_R/
#Book (Springer): https://www.imbi.uni-freiburg.de/lehre/lehrbuecher/meta-analysis-with-r
#Cochrane: https://handbook-5-1.cochrane.org/
#Data:
https://docs.google.com/spreadsheets/d/1otr6z3pLNdANC2YhEnL7bbdKl6j0Yisb7c78ATaKe9
0/edit#gid=1060103591
#PRISMA: http://prisma-statement.org/
#Key Reference: Cooper, Hedges, & Valentine: https://libcat.biola.edu/record=b2191233
#Hedges, Larry & Ingram Olkin (1985); Statistical Methods for Meta-Analysis
#Reference: https://www.researchgate.net/publication/325486099_How_to_Conduct_a_Meta-
Analysis_of_Proportions_in_R_A_Comprehensive_Tutorial

######################################################
##### Load Data
######################################################
library(readr)
All <- read_csv("Meta-Analysis_All-Cases.csv")
Severe <- read_csv("Meta-Analysis_Severe.csv")
Critical <- read_csv("Meta-Analysis_Critical.csv")
Mortality <- read_csv("Meta-Analysis_Mortality.csv")
Locations <- read_csv("Meta-Analysis_Locations.csv")


#######################################################
##### Run Models
#######################################################
##### For our data:
library(meta)
#settings.meta(plotwidth="3cm", fontsize=11)
pw = "7.5inch"
fs = 11

m.All <- metaprop(event=male,
        n=n,
        data=All,
        studlab=paste(Author),  #Author is their author column name
```

```
                comb.fixed = FALSE,
                comb.random = TRUE,
                #method.tau = "SJ",
                hakn = FALSE,
                prediction=FALSE,
                method.ci = "AC",
                sm="PRAW",
                plotwidth=pw, fontsize=fs)

m.Severe <- metaprop(event=male,
                n=n,
                data=Severe,
                studlab=paste(Author),  #Author is their author column name
                comb.fixed = FALSE,
                comb.random = TRUE,
                #method.tau = "SJ",
                hakn = FALSE,
                prediction=FALSE,
                method.ci = "AC",
                sm="PRAW",
                plotwidth=pw, fontsize=fs)

m.Critical <- metaprop(event=male,
                n=n,
                data=Critical,
                studlab=paste(Author),  #Author is their author column name
                comb.fixed = FALSE,
                comb.random = TRUE,
                #method.tau = "SJ",
                hakn = FALSE,
                prediction=FALSE,
                method.ci = "AC",
                sm="PRAW",
                plotwidth=pw, fontsize=fs)

m.Mortality <- metaprop(event=male,
              n=n,
              data=Mortality,
              studlab=paste(Author),  #Author is their author column name
              comb.fixed = FALSE,
              comb.random = TRUE,
              #method.tau = "SJ",
              hakn = FALSE,
              prediction=FALSE,
              method.ci = "AC",
              sm="PRAW",
```

```
        plotwidth=pw, fontsize=fs)  #Note: I should examine 'PLOGIT', 'PAS', 'PFT', 'PLN', to
make sure results are similar

# #Examine 'PLOGIT', 'PAS', 'PFT', 'PLN', to make sure results are similar
# m2 <- update(m1, sm='PLOGIT')
# m3 <- update(m1, sm='PAS')
# m4 <- update(m1, sm='PFT')
# m5 <- update(m1, sm='PLN')


### Display Output
m.All
m.Severe
m.Critical
m.Mortality

### Confidence Interval Plots
forest(m.All, leftlabs = c('Study', 'Males', 'Total'))
forest(m.Severe, leftlabs = c('Study', 'Males', 'Total'))
forest(m.Critical, leftlabs = c('Study', 'Males', 'Total'))
forest(m.Mortality, leftlabs = c('Study', 'Males', 'Total'))


##################################################################
##### Bias Results: Funnel Plots
##################################################################
### Install dmetar package
# if (!require("devtools")) {
#   install.packages("devtools")
# }
# devtools::install_github("MathiasHarrer/dmetar")

### Set up for colored points in funnel plots
#Added a 'Region' variable to the data with number of region, will call in funnel()
Region = c('China', 'Other Asia', 'Europe', 'USA') #Regions, coded 1,2,3,4
Color = c('red','dark gray','orange','blue')
# index = c(1,3,4,2,1,1) #Check
# Region[index]         #Check

library(dmetar) #For Egger's test

funnel(m.All,xlab = "Hedges' g", col=Color[All$Region], pch=19)
legend('topleft', legend = c('China', 'Other Asia', 'Europe', 'USA'),
    fill=c('red','dark gray','orange','blue'),
    title='Study Region')
title('Overall Cases')
```

```
#funnel(m.All,xlab = "Hedges' g",studlab = TRUE)
#title('Overall Cases')
eggers.test(x = m.All)

funnel(m.Severe,xlab = "Hedges' g", col=Color[Severe$Region], pch=19)
legend('topleft', legend = c('China', 'Other Asia', 'Europe', 'USA'),
      fill=c('red','dark gray','orange','blue'),
      title='Study Region')
title('Severe Cases')
# funnel(m.Severe,xlab = "Hedges' g",studlab = TRUE)
# title('Severe Cases')
eggers.test(x = m.Severe)

funnel(m.Critical,xlab = "Hedges' g", col=Color[Critical$Region], pch=19)
legend('topleft', legend = c('China', 'Other Asia', 'Europe', 'USA'),
      fill=c('red','dark gray','orange','blue'),
      title='Study Region')
# funnel(m.Critical,xlab = "Hedges' g",studlab = TRUE)
title('Critical Cases')
eggers.test(x = m.Critical)

funnel(m.Mortality,xlab = "Hedges' g", col=Color[Mortality$Region], pch=19)
legend('topleft', legend = c('China', 'Other Asia', 'Europe', 'USA'),
      fill=c('red','dark gray','orange','blue'),
      title='Study Region')
title('Mortality Cases')
# funnel(m.Mortality,xlab = "Hedges' g",studlab = TRUE)
# title('Mortality Cases')
eggers.test(x = m.Mortality)


###############################################################
##### Groups: China & non-China
###############################################################
### Function for inputting data and calling forest
forest.plot.region <- function(model, region, label, Asia=TRUE)
{
  index = 1:length(region)
  if(Asia==TRUE) region.index = ifelse(region==1 | region==2, index, NA)
  else region.index = ifelse(region==3 | region==4, index, NA)
  region.index = na.omit(region.index)

  m1 = update(model, subset=region.index)
  print(label)
  forest(m1, leftlabs = c('Study', 'Males', 'Total'))
}
```

```
forest.plot.region(m.All, region=All$Region, label='All, Asia', Asia=TRUE)
forest.plot.region(m.All, region=All$Region, label='All, West', Asia=FALSE)
forest.plot.region(m.Severe, region=Severe$Region, label='Severe, Asia', Asia=TRUE)
forest.plot.region(m.Severe, region=Severe$Region, label='Severe, West', Asia=FALSE)
forest.plot.region(m.Critical, region=Critical$Region, label='Critical, Asia', Asia=TRUE)
forest.plot.region(m.Critical, region=Critical$Region, label='Critical, West', Asia=FALSE)
forest.plot.region(m.Mortality, region=Mortality$Region, label='Mortality, Asia', Asia=TRUE)
forest.plot.region(m.Mortality, region=Mortality$Region, label='Mortality, West',
Asia=FALSE)

m1 = update(m.Critical, subset=c(4,6,7,8))
funnel(m1,xlab = "Proportion of Males", col=Color[c(3,3,4,3)], pch=19)
legend('topleft', legend = c('China', 'Other Asia', 'Europe', 'USA'),
      fill=c('red','dark gray','orange','blue'),
      title='Study Region')
# funnel(m.Critical,xlab = "Proportion of Males",studlab = TRUE)
title('Critical Cases in the West')
eggers.test(x = m1)

m1 = update(m.Critical, subset=c(1,2,3,5,9))
funnel(m1,xlab = "Proportion of Males", col=Color[1], pch=19)
legend('topleft', legend = c('China', 'Other Asia', 'Europe', 'USA'),
      fill=c('red','dark gray','orange','blue'),
      title='Study Region')
# funnel(m.Critical,xlab = "Proportion of Males",studlab = TRUE)
title('Critical Cases in the Asia')
eggers.test(x = m1)

#---------------------------------------------------------
##### Table by region
#---------------------------------------------------------
Region = c(All$Region, Severe$Region, Critical$Region, Mortality$Region)
Group = c(rep('All',23), rep('Severe',8), rep('Critical',9), rep('Mortality',6))
table(Group,Region)

#---------------------------------------------------------
##### Test by region
#---------------------------------------------------------
Male = c(45+11+22+62+19, 1304, 90, 15, 27)
Total = c(67+13+36+100+22, 1591, 124, 24, 48)
Female = Total-Male
data = rbind(Male,Female)
colnames(data) = c('China','Italy','France','USA','Spain')
chisq.test(data)
```

```
################################################################
##### Ages
################################################################
### Task
#a.	Fit flipped exponential with mean & SD, then find median
#   By 'flipped exponential, I mean instead of P(X) decreasing as x increases,
#   with an asymptote at 0, I mean P(X) increases as x increases,
#   with an asymptote at 90 or 100
#b.	Construct plot of median ages with 95% CI

### Data
xbars = c(38.8, 42.5, 45.1, 46.1, 52.7, 55.5, 57.6, 63.2, 64, 65.8, 70.7)
sds = c(13.8, NA, 13.35, 15.42, 15.5, 13.1, 13.7, 12, 18, 14.2, 10.9) #Note: 2nd element is
missing
#Need to impute an estimate of sd for the study with xbar=42.5
lm1 = lm(sds~xbars)
lm1$coefficients[1] + lm1$coefficients[2]*42.5 #14.48 is interpolated sd from regression
sds = c(13.8, 14.5, 13.35, 15.42, 15.5, 13.1, 13.7, 12, 18, 14.2, 10.9) #Note: 2nd element is
interpolated


#---------------------------------------------------------------
##### Distribution Fitting
#---------------------------------------------------------------
### Negative Binomial (p works out to be same as for geometric, leaving r as tuning
parameter...)
#Check possible values of r for tuning - I think we have it!
for (r in 5:25)
{
x = rnbinom(10000, size=r, prob=.27)
print(c(r, round(mean(x),1), round(sd(x),1)))
}

###Using MOM, we obtain phat = mean/var; rhat = mean*phat/(1-phat)
phat = xbars/sds^2
rhat = xbars*phat/(1-phat)

medians = qnbinom(0.5, size=rhat, prob=phat) #*** This is the key line obtaining the medians

i=11
x=0:100
px = dnbinom(x,size=rhat[i],prob=phat[i])
plot(x,px,type='h')

convert.mean.to.median <- function(xbar, sd, quantile=0.5)
{
```

```
  phat = xbar/sd^2
  rhat = xbar*phat/(1-phat)
  median = qnbinom(quantile, size=rhat, prob=phat)
  return(median)
}
convert.mean.to.median(58.2,15)


#-------------------------------------------------------
##### Generate Barplots
#-------------------------------------------------------
### Steps to take, for each group:
#1. Extract means & sds from group
#2. Obtain medians
#3. Combine medians into one vector, with sample sizes -> metamedian package
#4. Infer grand median                      -> metamedian package
#5. Calculate 95% CI                        -> metamedian package
#6. Barplots

###1. Get means for each group
Am = All$Age
Am[4]=55; Am[8]=45; Am[10]=44; Am[13]=41; Am[14]=57; Am[15]=51; Am[23]=37

Sm = Severe$Age
Sm[5] = convert.mean.to.median(58.2,15)

Cm = Critical$Age
Cm[8] = convert.mean.to.median(63.2,12)
Cm[7] = convert.mean.to.median(64,18)
Cm[9] = NA

Mm = Mortality$Age
Mm[1] = convert.mean.to.median(70.7,10.9)
Mm[3] = 72 #Fit failed, insert estimate
Mm[5] = convert.mean.to.median(65.8,14.2)


###2. Obtain medians [Stop.  Use library(metamedian)]

### 3. Use metamedian package to get CIs
library(metamedian)
All.med.CI = pool.med(yi=Am, wi=All$n, norm.approx=TRUE)
Severe.med.CI = pool.med(yi=Sm, wi=Severe$n, norm.approx=TRUE)
Critical.med.CI = pool.med(yi=Cm, wi=Critical$n, norm.approx=TRUE)
Mortality.med.CI = pool.med(yi=Mm, wi=Mortality$n, norm.approx=TRUE)
```

```
### 6. Barplots
middle = c(All.med.CI$pooled.est, Severe.med.CI$pooled.est, Critical.med.CI$pooled.est,
Mortality.med.CI$pooled.est)
lower = c(All.med.CI$ci.lb, Severe.med.CI$ci.lb, Critical.med.CI$ci.lb-1,
Mortality.med.CI$ci.lb)
upper = c(All.med.CI$ci.ub, Severe.med.CI$ci.ub, Critical.med.CI$ci.ub+1,
Mortality.med.CI$ci.ub)
group = c('All','Severe','Critical','Mortality')
group = factor(group, levels=c('All','Severe','Critical','Mortality'))
CI.data = data.frame(group,middle,lower,upper)

library(ggformula)
library(extrafont)

#Option 3b
gf_errorbar(lower + upper ~ group, width=0.1, size=1.5, data=CI.data) %>%
  gf_point(middle ~ group, data=CI.data, size=4) %>% #Add the error bars
  gf_lims(y = c(0, 80)) %>%
  gf_labs(x = "COVID-19 Severity",
          y = "Age (years)") +
  theme_bw(base_family = "Arial", base_size=28) +
  theme(
    text = element_text(face='bold'),
    axis.title = element_text(face='bold'),
    axis.line = element_line(size = 3))

#-----------------------------------------------------------
##### Test for difference of medians
#-----------------------------------------------------------
medians = c(Am, Sm, Cm, Mm)
group = c(rep('All',23), rep('Severe',8), rep('Critical',9), rep('Mortality',6))
lm2 = lm(medians ~ group)
anova(lm2)
plot(lm2) #pretty non-normal residuals, better use kruskal.test()
kruskal.test(medians ~ group) #p-value = 2.416e-05, still radical reject
```
