# Supplementary material for: Systematic Review and Meta-Analysis of Sex-Specific COVID-19 Clinical Outcomes
Source: Front Med (Lausanne). 2020 Jun 23;7:348. doi: 10.3389/fmed.2020.00348 (PMC7331754; doi:10.3389/fmed.2020.00348)
Supplement: Supplementary file 2 [file Table_1.PDF]

Systematic Review and Meta-Analysis of Sex-Specific COVID-19 Clinical Outcomes

Supplemental Table 1

Database Search Terms and Research Articles Identified

Search Date: 04/16/2020 (All published articles up to 04/16/2020)

Search Terms	LitCOVID (PubMed)	Web of Science (WoS)	OVID (Embase)
All COVID-19 Articles	4929	4068	2916
Male	103	193	42
Female	66	168	25
Men	41	55	27
Women	124	112	62
Sex	28	36	23
Gender	24	23	13
Total Search Results	386	587	192
After Removing Duplicates	281	376	129

Combined total articles from all three databases: 786

Number of unique articles after removing duplicates: 414
